# Tumor microenvironment in glioblastoma: Current and emerging concepts

**DOI:** 10.1093/noajnl/vdad009

**Published:** 2023-02-23

**Authors:** Pratibha Sharma, Ashley Aaroe, Jiyong Liang, Vinay K Puduvalli

**Affiliations:** Department of Neuro-Oncology, The University of Texas MD Anderson Cancer Center, Houston, Texas, USA; Department of Neuro-Oncology, The University of Texas MD Anderson Cancer Center, Houston, Texas, USA; Department of Neuro-Oncology, The University of Texas MD Anderson Cancer Center, Houston, Texas, USA; Department of Neuro-Oncology, The University of Texas MD Anderson Cancer Center, Houston, Texas, USA

**Keywords:** cell–cell communication, electrical coupling, exosome, extracellular matrix, glioblastoma, paracrine signaling, tumor microenvironment

## Abstract

Glioblastoma (GBM) tumor microenvironment (TME) is a highly heterogeneous and complex system, which in addition to cancer cells, consists of various resident brain and immune cells as well as cells in transit through the tumor such as marrow-derived immune cells. The TME is a dynamic environment which is heavily influenced by alterations in cellular composition, cell-to-cell contact and cellular metabolic products as well as other chemical factors, such as pH and oxygen levels. Emerging evidence suggests that GBM cells appear to reprogram their the TME, and hijack microenvironmental elements to facilitate rapid proliferation, invasion, migration, and survival thus generating treatment resistance. GBM cells interact with their microenvironment directly through cell-to-cell by interaction mediated by cell-surface molecules, or indirectly through apocrine or paracrine signaling via cytokines, growth factors, and extracellular vehicles. The recent discovery of neuron–glioma interfaces and neurotransmitter-based interactions has uncovered novel mechanisms that favor tumor cell survival and growth. Here, we review the known and emerging evidence related to the communication between GBM cells and various components of its TME, discuss models for studying the TME and outline current studies targeting components of the TME for therapeutic purposes.

Glioblastoma (GBM) is a highly treatment resistant primary brain tumor with few effective treatment options.^1^ Despite multimodality therapy, these tumors unfailingly recur due to intrinsic and adaptive resistance, tumor heterogeneity, and immune evasion.^2^ The tumor microenvironment (TME) niche is comprised of endothelial cells, neurons, astrocytes, oligodendrocytes, resident immune cells such as microglia, tumor-infiltrating circulating immune cells such as tumor-associated macrophages and tumor-infiltrating lymphocytes (TILs), and noncellular components such as apocrine and paracrine signaling molecules, exosomes, extracellular matrix (ECM) components, and secreted ECM remodeling enzymes.^3^ Due to its diverse components and dynamic nature, the TME plays a vital role in the survival of cancer cells and their response to therapy.^4^

Glioma cells take a central role in regulating the functions of cellular and noncellular components of the TME via complex signaling networks which enables them to regulate processes such as biomass synthesis, maintenance of cellular processes and resistance to therapies that facilitates their survival.^5,6^ The communication between tumor and surrounding cells is achieved by soluble factors such as cytokines, chemokines, matrix remodeling enzymes, and growth factors. In addition to these mechanisms, tumor cells are known to employ exosomes, gap junctions, circulating tumor cells, tunneling nanotubes, cell-free DNA, and horizontal DNA transfer to interact with other tumor or normal cells.^7–9^ In addition, the recognition of the essential nature of the TME and the complex communication network between tumors and normal cells for GBM development and progression has yielded a new focus for therapeutic targeting of GBM.^9–12^

The focus on the GBM development of novel therapies targeting the TME has also raised the need for reliable models that recapitulate the TME in patients. In addition to conventional rodent intracranial xenograft models, newer genetically engineered and humanized rodent models have been developed to address specific questions related to the TME; additionally, more recent development of advanced 3-dimensional tumor models have helped further bridge the gap between basic discoveries and their translation through therapeutic modeling of GBM.^13,14^

In this review, we discuss the components of GBM TME and their interactions with the tumor cells and among one another (Figure 1). We also discuss the mechanisms used by local and distant GBM cells to interact with the surrounding normal cells and examine the relevance of current preclinical models available to study TME in GBM. The topic is broadly categorized into immune, nervous system, and chemical components in the following sections.

**Figure 1.**
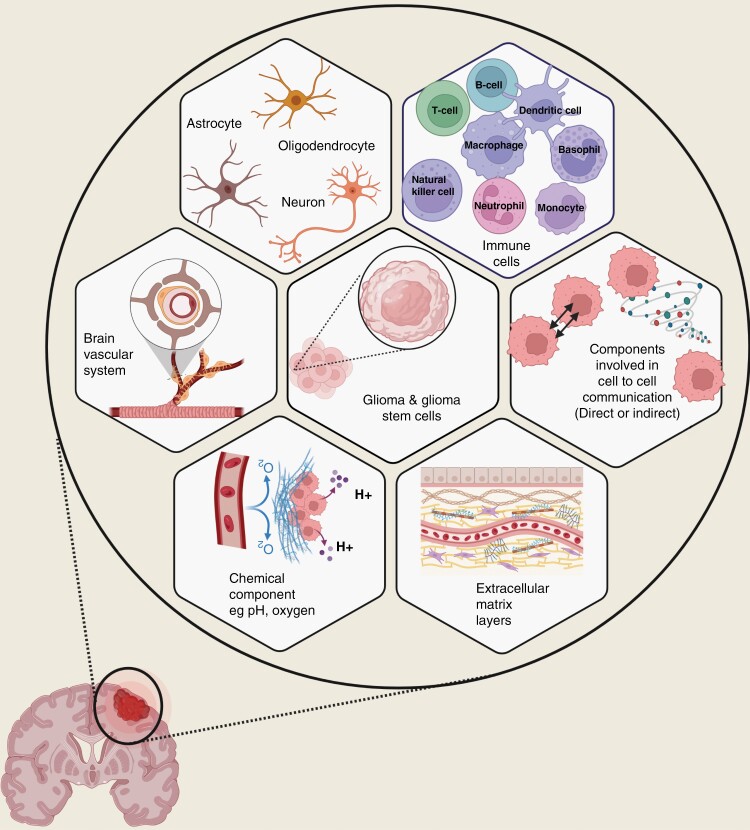
Schematic representation of the glioma tumor microenvironment components. The glioma tumor microenvironment is a complex, heterogeneous, and interactive system that is consisted of glioma and glioma stem cells, immune cells, nervous system, brain vascular system, and extracellular matrix layers. The factors involved in direct and indirect cell communication and chemical tumor microenvironment such as pH and oxygen also play a significant role in modulating glioma tumor microenvironment.

## Immune Component

Emerging knowledge of the tumor-related immune system has led to novel therapeutic approaches and immunotherapies including immune checkpoint inhibition (ICI) that has revolutionized the management of a growing list of cancer types. Newer methods of immune therapy including chimeric antigen receptor (CAR) T cells, vaccine-based approaches and oncolytic virus-based therapies are under rigorous investigation. While the development of such approaches has seen a significant impact on survival of patients in other cancers, the same has not been seen in GBM patients due to multiple limitations that are unique to brain cancer including its characteristic heterogeneity, low immunogenicity, a profound immune suppressive microenvironment, and the blood–brain barrier (BBB) that restricts immune infiltration. In this section, we review recent findings regarding the mechanisms underpinning anti-glioma immunology with a focus on the major immune components of the glioma microenvironment and an emphasis on data from human studies.

The central nervous system (CNS) is historically considered as an immune-privileged organ guarded by the BBB where adaptive immunity and inflammatory response are tightly controlled. However, this concept has been recently challenged with the discovery of the existence of a glymphatic system (the meningeal lymphatic compartment) and lymphatic CNS drainage to cervical lymph nodes, forming a foundation for adaptive immunity associated with CNS inflammation and certain neurodegenerative diseases.^15,16^ This significant progress has provoked speculation of a role for the meningeal lymphatic system in glioma immune response and has been demonstrated subsequently in mouse models of brain tumors.^17,18^ However, the meningeal lymphatic compartment is not directly connected to the brain parenchyma, and the relevance of its role in glioma immunity in humans awaits further study. Indeed, extracranial metastases of primary gliomas are rare and lymphatic spread is extremely rare.^19^ In addition to the meningeal lymphatic system, tertiary lymphatic structures (TLSs) have been found to exist in brain tumors.^20^ TLSs are alternative sites for T-cell priming and the only known routes of lymphocyte infiltration, which may permit glioma immune responses and warrant further studies. The CNS relies heavily on innate mechanisms that are mediated by neurons, astrocytes, and resident immune cells, mainly microglia, for immune defense against pathogens. This distinct version of immune defense has a clear impact on the glioma microenvironment where immune cells, which can constitute up to 50% of the tumor cellularity, consist of microglia, glioma-associated macrophages (GAMs), and, less abundantly, monocytes, neutrophils, and TILs.^21–23^ These innate and adaptive immune cells and inflammatory infiltrates co-exist with cancer cells, neurons, multiple lineages of glial cells, and vascular endothelial cells, which shape the glioma immune microenvironment through direct and indirect interactions. Recent studies, as facilitated by single-cell approaches, among others, have begun to paint a complex picture of an interplay of tumor, immune, and host cells, which is conducive to a profound immunosuppressive microenvironment. This scenario can be further exacerbated by metabolic stress, hypoxia, and damage-associated molecular patterns that are common in high-grade gliomas and GBMs. While the immune suppressive microenvironment plays an important role in thwarting the launch of anti-glioma immunity and therapeutic immune responses, most gliomas are intrinsically poorly immunogenic across the cancer continuum, adding an additional hurdle for immunotherapy. As such, therapeutic interventions may benefit from a dual strategy exploiting glioma immunogenicity and circumventing the immunosuppressive glioma microenvironment.

### Microglia and Macrophages

Microglia and GAMs are the most abundant immune cells in the microenvironment of most primary gliomas, pertaining to the CNS milieu. As such, these myeloid cell lineages have been the focus of extensive investigations, which have demonstrated a remarkable origin, phenotype, and functional diversity and plasticity. Microglia and some of the macrophage populations are derived from embryonic progenitors in yolk sac, whereas bone marrow-derived macrophages are believed to originate from peripheral monocytes that infiltrate the TME as in other pathological conditions. Additionally, new findings in mice showed a meningeal pool of monocytes and neutrophils that were supplied directly from the adjacent bone marrow of the skull, which could reach the brain parenchyma under pathological conditions of CNS injuries and inflammation.^24^ It remains unclear to what extent the same route of infiltration operates in brain tumors.

The glioma microenvironment can have a significant impact on microglia and GAMs,^23^ which constitute highly dynamic cell populations. As demonstrated extensively in cancer as well as noncancer contexts, different cytokine and chemokine conditions can induce macrophages to undergo either M1 or M2 polarization with M1 macrophages exhibiting immune-supportive and cancer-inhibitory functions, as opposite to M2 macrophages. Although this conventional phenotyping may not adequately cover the functional complexity of GAMs,^25^ these myeloid lineages are generally immunosuppressive in the glioma microenvironment. Although the underlying mechanisms are not fully understood, GAMs can produce anti-inflammatory cytokines (IL4, IL10, and TGFβ) and tumor-promoting factors (IGF-1, EGF, and PDGF), promote angiogenesis (VEGF and IL8), disrupt metabolism (ARG1- and IDO-mediated amino acid depletion), and activate immune checkpoints by expressing PD-L1 and CD39.^26–28^ Given the overwhelming abundance of GAMs, which are professional cells that perform phagocytosis (ie, engulfment of dead cells, pathogens, and debris, and antigen presentation), it is tempting to develop therapeutic strategies based on harnessing some of these cell populations. One such approach targets the colony-stimulating factor 1 receptor (CSF1R) because glioma cells produce the ligand cytokines CSF1 and IL34 abundantly, which act on GAMs through CSF1R. Several CSF1R inhibitors have been developed that are effective in reprogramming GAMs toward an antitumor phenotype, reducing M2 polarization, inhibiting tumor growth, and increasing survival in a murine GBM model in multiple preclinical settings.^29,30^ However, a phase II study of PLX3397, a CSF1R inhibitor, conducted to treat patients with recurrent GBM failed to show therapeutic efficacy despite the excellent tolerance and blood–tumor barrier penetration capacity and on-target drug action.^31^ Additional preclinical studies and clinical trials have also been conducted to test other CSF1R inhibitors, such as BLZ945, pexidartinib, and blocking antibodies, either as single agents or in combination with radiotherapy or ICI.^32–35^ In brain cancer models, however, as demonstrated in mice, following initial therapeutic benefit, CSF1R inhibitor-treated tumors recur frequently driven by elevated macrophage-derived insulin-like growth factor 1 (IGF-1) and high IGF-1 receptor (IGF-1R) levels on tumor cells and other mechanisms.^36,37^ Further, unlike in other tumors, inhibition of CSF1R had minimal impact on CD8+ T-cell infiltration in a syngeneic mouse GBM model, highlighting the immunosuppressive microenvironment of brain tumors, and that CSF1R inhibition alone appears to be insufficient to elicit strong anti-glioma immunity.^38–40^

### Other Infiltrating Myeloid Cells

As found in many other cancer types, myeloid-derived suppressive cells (MDSCs), which are derivatives of monocytic or granulocytic cells, are also detected in the glioma TME. These cells suppress tumor-specific effector T cells in a manner similar to that of GAMs,^41,42^ although they are distinguished from GAMs and microglia based on the expression of different cell-surface markers. However, single-cell mRNA profiling^22,23^ failed to distinguish MDSCs from other myeloid cell lineages. Additional investigation of brain tumor-associated MDSCs at single-cell resolution with increased depth may reveal heterogeneous cell states and provide improved clarity. On the other hand, these single-cell studies showed the presence of abundant neutrophils in the glioma TME. Although neutrophils also appear to play a generally immunosuppressive role in GBM tumors, the role of this cell population is not well studied. Given their phagocytic and secretory activities and their unique ability to interact with tumor cells, neutrophils are likely to have a more important role in the glioma TME than has been demonstrated to date.

### Natural Killer Cells

Natural killer (NK cells, CD56^+^CD3^−^ cells) are potent effector lymphoid cells that mediate antigen-independent immune surveillance against pathogens and stressed cells through “missing-self” recognition, which can also perform innate antitumor immunity. In GBM, however, NK cells were found to be one of the least numerous immune cell populations of all tumor-infiltrating immune cells and were predominantly the CD56^dim^CD16^−^ subtype.^43^ Newer single-cell studies have confirmed the scarcity of NK cells in primary gliomas with identification of CD16^−^ immature NK cells in IDHwt tumors, whereas CD16^+^ cytotoxic NK cells are present in IDH1mut tumor and brain metastases.^22^ Further, NK cells are inhibited by classic (antigen-presenting) and nonclassic HLA-I molecules through binding to the inhibitory killer immunoglobulin-like receptors. In gliomas, although HLA-I genes are subject to loss of heterozygosity, complete deletion of all of these genes is uncommon. In addition, the glioma TME plays an important role in suppressing NK cell function, since TGFβ, which is secreted by cancer and noncancer cells, downregulates the expression of NKG2D-activating receptor on NK cells isolated from GBM patients.^44^ Thus, the role of NK cells in anti-glioma surveillance remains to be demonstrated. Nonetheless, adoptive NK cells have been demonstrated to exhibit cytotoxic activities against glioma cells, including glioma stem cells, in preclinical settings, and targeting some of the inhibitory mechanisms was recently shown to increase NK cell function.^45–47^

### Impaired Lymphocyte Infiltration and Effector T-Cell Function

While T lymphocytes appear to be able to traffic into brain tumors, in contrast to the abundance of myeloid cells, primary gliomas are typically poorly infiltrated with lymphocytes, including NK and T cells, which constitute only a small proportion of the immune cells.^23^ Although CD8^+^ cytotoxic T cells and CD4^+^ helper T cells are found in GBM, T cells are often dysfunctional as a result of senescence, tolerance, anergy, or exhaustion.^48^ Further, conflicting observations have been made regarding the clinical significance of T-cell infiltration in gliomas.^3,43,49,50^ As such, to what extent glioma TILs can successfully launch antitumor immunity remains unclear. Multiple factors contribute to the immunosuppressive glioma TME and are conducive to the suppression of T-cell-mediated anti-glioma immunity. Recent studies also highlight the impact of tumor type on the local tissue microenvironment, with IDH1 mutant gliomas being associated with more profound immune suppression, compared with brain metastases which are populated with relatively more lymphocytes.^22,23^ In addition to the anatomical restrictions that impede T-cell trafficking to brain tumor parenchyma, bone marrow sequestration of lymphocytes in bone marrow was also shown to impair tumor infiltration in GBMs as a result of tumor-imposed internalization of the S1P receptor, S1P1, on T cells.^51^

Unlike many other cancer lineages, gliomas are poorly responsive to ICI, an immunotherapy modality that marks one of the most significant advances in cancer management. ICI counteracts the exhaustion phenotype of cytotoxic T cells expressing the immune checkpoint receptor PD1 and, therefore, it requires preexisting tumor antigen-specific T cells. While gliomas are in general resistant to ICI despite the remarkable genetic and epigenetic heterogeneity of the tumor lineages, brain metastases are more sensitive to immune checkpoint blockade with complete responses being achieved in some patients.^52–55^ These observations suggest that neither the brain TME nor tumor type alone dictates sensitivity to ICIs; instead, it appears to be a result of factors that are both intrinsic to cancer cells and related to the extracellular milieu. In addition to the notoriously immunosuppressive TME, gliomas are poorly immunogenic, representing a tumor type with lower tumor mutational burden (TMB) and hence a lower availability of immunogenic neoantigens and antigen-specific T-cell pools.^56,57^ In addition to TMB and the resultant nonsynonymous single (or double) nucleotide variants that give rise to potential neoantigens, several other mechanisms can produce immunogenic peptides and cancer-associated antigens, which have been the subject of emerging new studies, including genetic and epigenetic alterations, transcriptional and pre-mRNA splicing events, noncanonical translation, and post-translational modifications.^58–63^ Pan-cancer studies thus far have demonstrated that gliomas are less affected by these various tumor antigenic mechanisms as compared with most other cancers, consistent with an immunologically “cold” phenotype. Tumor-specific antigens undergo MHC-I or -II restriction for T-cell priming and recognition. Most neoantigen discovery and vaccine development studies focus on HLA-I restricted neoepitopes, which can be recognized by CD8^+^ effector and memory T cells enabling T-cell-mediated cytotoxicity. However, naive T cells must first be primed by dendritic cells (DCs), which access and process tumor antigens and then mediate HLA-I antigen presentation and co-stimulation normally observed in lymphoid organs. Somatic HLA-I loss of heterozygosity, a pan-cancer genetic aberration underlying the immune escape of cancer cells, affects ~10% of gliomas, which is less frequent than most other cancers.^64^ However, the brain parenchyma is largely devoid of appropriate lymphatic vessels limiting direct access to potential tumor antigens by DCs. Non-DC antigen-presenting cells, such as macrophages, process tumor antigens through the endocytic pathway and HLA-II complex and the effectiveness of this mechanism in eliciting anti-glioma immune response remains unclear despite the abundance of these cells in the glioma TME. Further, most GAMs are tumor promoting, and their antigen-presenting function is inhibited due to STAT3 activation.^65^ Instead, it is conceivable that the phagocytic phenotype of GAM due to M2 polarization may play a role in scavenging damaged tumor cells further reducing tumor antigen accessibility to professional antigen-presenting DCs. Indeed, although necrosis is a common phenotype of high-grade GBMs associated with reduced patient survival, these tumors remain poorly inflamed.

Given that insufficient immunogenicity and the immunosuppressive TME are the major hurdles limiting anti-glioma immunity, several immunotherapeutic strategies have been devised to increase glioma immunogenicity, including DC vaccines, STING agonists, oncolytic virus, and epigenetic modulators. The therapeutic effects of these approaches rely on elicitation of tumor antigen-specific immune responses and the cytotoxicity of effector T cells, providing a clear rationale for combination with ICI.^66,67^ Immunogenicity defects can also be bypassed by CAR T cells, which are engineered to express artificial tumor antigen ligands eliminating the priming step and the dependence on DC-mediated antigen presentation. The CAR T-cell approach has been demonstrated to be highly effective in B-cell malignancies and can be used in drug-resistant diseases, where these cells can remain therapeutically active in patients for an extended period of time.^68^ CAR T cells specific for IL13RA2, CD276, CD133, αvβ3 integrin, EGFR, and the oncogenic EGFRvIII variant that target GBM cells have been developed and tested in mouse models^69–71^ However, CAR T cells have demonstrated limited therapeutic efficacy in patients in clinical trials except for a recent report of prolonged survival achieved with EGFRvIII CAR in a patient with recurrent GBM.^72^ Effectiveness of CAR T-cell therapies in solid cancers, including gliomas, is confounded by the limitations of adequate distribution of these cells in the TME, low antigen coverage due to inter- and intratumoral heterogeneity, antigen escape, suppression by the TME, and failure to attain long-term persistence in recipients because of exhaustion, senescence, and cell death. The mechanisms of CAR T-cell dysfunction may also operate in other antigen-specific effector T cells, and some of the mechanisms are only beginning to be elucidated. For example, compelling evidence shows that CAR T cells can extract tumor antigen from target cells through a mechanism known as trogocytosis, which underlies multiple aspects of CAR T-cell function, including tumor antigen escape, fratricidal CAR T-cell death, and the triple exhaustion phenotype.^73^ Further, epigenetic reprogramming underpins the exhaustion phenotype in CAR as well as non-CAR T cells.^74–79^ In addition, cancer cell-intrinsic IFNγR pathway has been shown to be essential for CAR T-cell-mediated killing of GBM and other solid tumor cells but not for hematologic malignancies,^68^ suggesting that impaired IFNγR signaling may represent a cancer cell-intrinsic mechanism of resistance to CAR T cells. Therapeutic approaches targeting these mechanisms are currently being integrated into immunotherapy modalities based on ICI and CAR T cells.

## Nervous System Component

**Figure 2.**
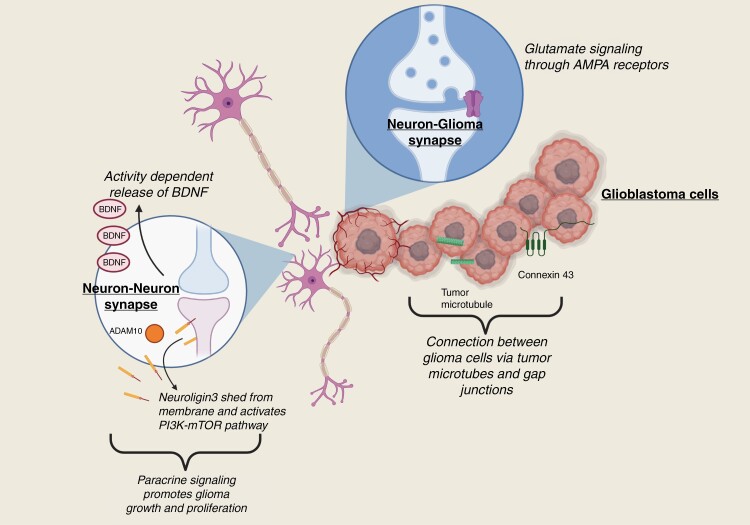
Relationship between chemical and biological factors influencing the glioma tumor microenvironment in the nervous system.

A growing area of interest is the potential for gliomas to communicate with neurons and glia. Interactions with the peripheral nervous system have been described in many tumors, including pancreatic, colorectal, and head and neck cancers, wherein sympathetic, parasympathetic, or sensory innervation influences tumor proliferation and invasiveness.^80^ The unique characteristics of both glioma cells and the native cells of the CNS facilitate even more complex means of interaction (Figure 2). Accumulating evidence suggests that bidirectional communication between the native nervous system cells and glioma cells can alter tumor growth kinetics and behavior. The mechanisms through which this communication occurs and explore potential therapeutic implications are under intense ongoing study.

As first observed by Santiago Ramón y Cajal, glial cells tend to cluster around neurons. This phenomenon was further characterized in the 1930s and has been termed perineuronal satellitosis.^81^ Recapitulating this interaction, glioma cells also exhibit a tendency toward perineuronal satellitosis.^82^ It has since been demonstrated that neurons may interact with glioma cells through paracrine stimulation, synaptic transmission, and neurotransmitters, as well as other indirect means.

The study of paracrine interactions between the nervous system and glioma cells has centered on the roles of brain-derived neurotrophic factor (BDNF) and neuroligin 3 (NLGN3). BDNF (also called abrineurin or neurotrophin), is a molecule that has been implicated in neuronal growth, differentiation, and apoptosis via signaling through the transmembrane tyrosine kinase B (TrkB) receptor (NTRK2).^83^ BDNF exists in immature (proBDNF) and mature forms, both of which play crucial roles in both early development of the nervous system and modulation of synaptic plasticity in adulthood. BDNF and TrkB expression are increased in cancers of various types, including gliomas.^84,85^ Intrinsic expression of BDNF and TrkB has been noted in C6 glioma cells.^86^ and exogenous exposure of these cells to recombinant murine mature BDNF increased proliferation, where antibody-mediated blocking of the BDNF abrogated these effects in a dose-dependent fashion. Additionally, glioma cells had increased motility and invasion in the presence of BDNF, as well as decreased rates of apoptosis. The balance in proportions of proBDNF and mature BDNF, which appear to exert opposing functions in cell growth, appears to correlate with tumor grade in glioma.^87^ These studies suggest that targeting mature BDNF signaling may represent a potential therapeutic strategy against gliomas. Of note, given the delicate homeostatic balance between BDNF and proBDNF expression, as well as the important roles these proteins play in the function of the nervous system and development of neurologic disease (including depression,^88^ Parkinson disease,^89^ dementia,^90^ and schizophrenia^91^), agents to block BDNF signaling, such as NTRK inhibitors, could potentially carry the risk of neurological toxicity and additional studies are needed to balance the risks and benefits of targeting the BDNF pathway.

NLGN3 is a member of the neuroligin family of cell adhesion molecules which regulate synaptic structure, plasticity, and function; it is expressed in both excitatory and inhibitory neurons as a postsynaptic transmembrane protein and NLGN3 alterations can alter synaptic function.^92,93^ Based on the hypothesis that neuronal activity has a mitogenic effect on glioma cells, 1 study revealed that soluble NLGN3 activated the PI3K–mTOR pathway in human glioma cell lines.^94^ Beyond promoting cell proliferation and growth through the previously characterized mechanisms of the PI3K–mTOR pathway, NGLN3-mediated PI3K–mTOR activation also resulted in the unexpected feed-forward secretion of additional NLGN3. Optogenetic studies utilizing light-mediated modulation of neuronal activity in vivo specifically within deep cortical neurons expressing channelrhodopsin-2 in the premotor cortex in a mouse orthotopic xenograft model of pediatric GBM have demonstrated a correlation between neuronal activity and glioma proliferation which was mediated by NGLN3. Conversely, NLGN3 depletion had an adverse effect on proliferation, although this effect was only partial suggesting a role for other mitogens such as BDNF.

Subsequent studies identified that NLGN3 is cleaved by ADAM10 and secreted into the TME 0and ADAM10 inhibitors were found to inhibit the release of NLGN3 and consequently prevent growth of patient-derived orthotopic xenograft.^95^ In addition, *Nlgn3* knockout mice are unable to harbor patient-derived orthotopic xenografts of several molecular subtypes of high-grade glioma and show durable inhibition of glioma growth despite minimal impact on neurologic function (attributed to the increased compensatory expression of other neuroligins in normal synapses). The important role of the PI3K–mTOR pathway was confirmed, with the additional discovery of focal adhesion kinase-mediated downstream effects.

Additional evidence of BDNF and NLGN3 involvement in glioma is reflected in the discovery that photic stimulation can modulate the growth of glioma cells, specifically in mouse models of neurofibromatosis type 1 with optic pathway glioma.^96^ Light deprivation, achieved by rearing mice in the dark during weeks when tumors are normally expected to develop, decreased the formation and growth of optic pathway gliomas. Optogenetic promotion of optic pathway glioma growth was mediated in these low-grade gliomas by BDNF and NLGN3 secretion, which in turn was promoted by retinal activity. A direct link between neuronal activity and gliomagenesis was reported through stimulation of a unilateral optic nerve in mouse models of optic pathway glioma, also expressing channelrhodopsin-2 in retinal ganglion cells. The optic nerves in blue-light stimulated mice were larger and exhibited a higher proportion of glial or glioma cells. It should be highlighted that the effects of NLGN3 appeared to be similar across different histologic and molecular subtypes of glioma, at least in these models. The *NF1* mutation, synergistically with optogenetic stimulation of the retina, has been shown to contribute to NLGN3 secretion and optic glioma growth and further drives neuronal hyperexcitability and tumor progression through altered hyperpolarization-activated cyclic nucleotide-gated channels.^97^ In a similar context, sensory input of other types impacts glioma formation as well, particularly in the olfactory bulb wherein olfactory receptor neurons were shown to excite mitral and tufted cells into releasing IGF-1, thereby promoting gliomagenesis.^98^

Beyond the effects of soluble synaptic proteins, the synapse itself has a unique structure that may affect glioma cells via direct electrochemical signaling from neurons. Some glioma cells robustly express synaptic genes and form functional connections with neurons. Signaling from neurons was shown in work by Venkatesh et al. to induce AMPA (glutamate) receptor-mediated depolarization and excitatory postsynaptic potentials in glioma cells, which could be abolished with the sodium channel blocker tetrodotoxin.^99^ The gap junctions between glioma cells themselves also were noted to facilitate broad propagation of these impulses throughout the tumor, demonstrated through experiments including the administration of gap junction blocking agents which reduced the amplitude of glioma currents.

Connectivity via gap junctions was also reflected in studies revealing that spontaneous slow inward currents in neuron–glioma co-cultures increased input resistance and similarly could be blocked by gap junction blocking agents.^100^ These studies build on the work by Osswald et al. and who characterized “tumor microtubes” or protrusions emanating from the membranes of human astrocytoma cells, and first demonstrated the ability of these structures to propagate intracellular calcium waves via gap junctions.^101^ More recently, Venkataramani et al. found that these currents represented a functional connection both among glioma cells and between them and local astrocytes, and further demonstrated that neuronal activity increased tumor microtubule turnover and accelerated invasive behavior.^102^ Knockdown studies of the gene encoding connexin 43, a gap junction protein, in both in vitro and in vivo models further emphasize the importance of such junctions on intratumoral cell communication and glioma progression.^103^

Glutamate-mediated signaling may contribute to feedback loops resulting in glioma–neuron network hyperexcitability, as glioma cells also release glutamate into the surrounding tissue.^104,105^ The mechanism through which they may do so is thought to largely depend on increased expression of a transporter that exchanges extracellular cysteine for glutamate.^106^ The resulting increase in neuronal activity may contribute to seizure risk and invasiveness.^106,107^ Despite this, clinical trials of agents targeting glutamatergic neurotransmission have not shown significant benefit to date.^108^

The way in which synaptic transmission affects glioma cells is an area of active investigation; beyond glutamatergic transmission, glioma cells also express functional serotonin, GABA, and dopamine receptors and are therefore potentially vulnerable to the effects of these neurotransmitters.^109,110^ There is conflicting evidence regarding the potential impact on gliomas of antidepressants that modulate serotonergic tone; glioma incidence is apparently decreased in patients who have taken tricyclic antidepressants on a long-term basis,^111,112^ but not in those taking selective serotonin reuptake inhibitors, and there is no association between selective serotonin reuptake inhibitor use and survival in GBM.^113^ Tricyclic antidepressants may act via other broad neurotransmitter effects or separate mechanisms. Blocking dopamine receptor D4 has been shown to inhibit glioma cell proliferation,^114^ and ONC201, a small molecule used to treat diffuse midline glioma, works to block the dopamine receptors DRD2 and DRD3, in addition to inhibiting the phosphorylation of Akt and ERK. There may also be an emerging role of GABAergic neurotransmission in the electrical microenvironment. Glioma cells have been noted to upregulate expression of GABA receptors when in proximity to neurons,^115^ and GABAergic currents have been described in glioma cell lines.^116^ One study linked increase in endogenous GABA levels impede tumor growth, and further work in this field is ongoing.^117^

Neurons may be able to interact with cancer via indirect effects on the TME. It has been demonstrated that adrenergic nerves in the periphery are capable of modulating endothelial cell metabolism, with a demonstrable impact on angiogenesis in models of prostate cancer.^118,119^ The release of neurotransmitters and neuropeptides may also influence neo-angiogenesis. Neuronal activity is also known to affect immune function,^120^ although how such interactions might shape the glioma immune microenvironment specifically is less certain.^121^

Glial cells are also able to interact with tumor cells. Ameboid-appearing microglia, which were first described in association with glioma cells by Wilder Penfield in 1925, appear to promote glioma cell migration and proliferation.^122^ These microglia are described further as components of the immune microenvironment in the immunology section of this review. On histologic examination, gliomas are also commonly seen in association with reactive, large astrocytes.^123^ These astrocytes modulate glioma invasiveness, likely through the release of neurotrophic factors and increased production of matrix metalloproteases.^124^ Astrocytes may additionally link with glioma cells via gap junctions which, as in physiologic astrocyte–astrocyte gap junctions, mediate the exchange and distribution of toxins and small molecules across a wide network to promote cell health and homeostasis.^125^

## Chemical Component

**Figure 3.**
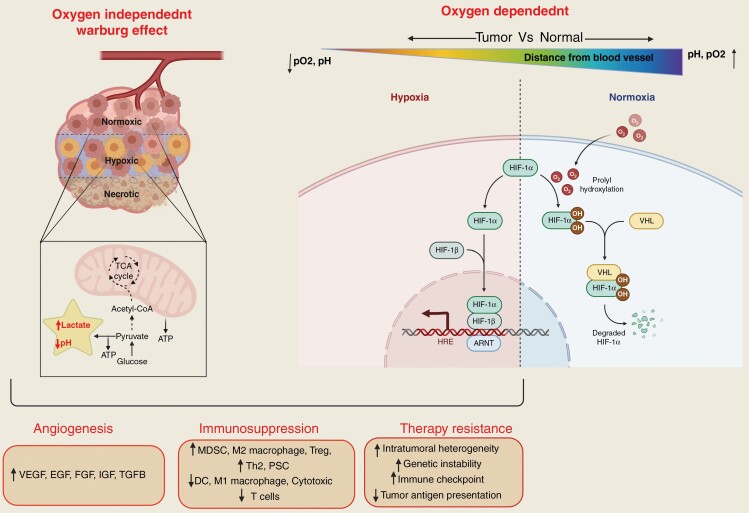
The role of oxygen dependent (HIF1-α mediated) and independent (Warburg effect), chemical tumor microenvironment in glioma angiogenesis, immunosuppression, and therapy resistance. ARNT, aryl hydrocarbon receptor nuclear translocator; EFG, epidermal growth factor; FGF, fibroblast growth factor; HIF1-α, hypoxia-inducible factor 1-α; HIF1-β, hypoxia-inducible factor 1-β; HRE, hypoxia response element; IGF, insulin-like growth factor; VEGF, vascular endothelial growth factor; VHL, Von Hippel–Lindau; TGFB, transforming growth factor β.

In addition to the concept of cancer being a disease of genetic and epigenetic alterations, several studies have shown a significant role for metabolic reprogramming interactions between stromal and tumor cells.^126,127^ Tumor cells adopt several aberrant metabolic processes to meet the energy demands associated with rapid proliferation and survival. For instance, glioma cells increase the uptake of glucose and other nutrients to support the aggressive biomass production in GBM. However, nutrient supply to the tumor can significantly vary due to the heterogeneity of the neoangiogenesis and the vascular network in GBM; such intratumoral variances in vascular supply can also alter the availability of oxygen within tumor, which in turn can influence the metabolic properties and energy utilization of cancer cells.^126,128,129^ Therefore, tumor cells within the same tumor can have differential metabolic signatures (Figure 3) with profound differences in physiological factors, such as extracellular pH and oxygen concentration between normal and cancer cells. These factors are linked to tumor progression, immunosuppression, therapy resistance, and metastasis.

### Tumor Acidosis

A major characteristic of developing malignancies is the emergence of tumor acidosis which progressively affects both intrinsic cellular processes as well as the TME which can help establish an immunosuppressive TME and confer treatment resistance.^130^ Several lines of evidence point to the tumor-specific advantages of an acidotic TME to tumor cells. For instance, factors responsible for acidosis include metabolic adaptations by the GBM cells such as a shift toward aerobic glycolysis (Warburg effect) which allows the cells to accumulate high levels of metabolic intermediates, resulting in lower extracellular TME glucose concentration, higher lactic acid and H^+^ production, and secretion, and changes in tumor energy utilization. Given that the lowering of intracellular pH may result in programmed cell death, cancer cells enhance their ability to secrete these metabolites into extracellular space through secretory mechanisms such as upregulated MCT-4 (monocarboxylated transporter) and Na^+^-H^+^ transporters^78,131–135^ and lowering the pH of the TME.^126,136^ The acidic environment in the TME can facilitate several other pro-tumorigenic processes including proliferation, migration, and angiogenesis. Also, given that such an acidic environment is a potential threat to cancer cells, to counter this, malignant cells activate protective mechanisms such as increasing the expression of proteins such as LAMP2 which can protect lysosomal and plasmalemmal membranes from acid proteolysis and increase expression of autophagy-related proteins such as ATG5, and anti-apoptotic proteins such as BCL-2 upon chronic exposure to low pH conditions; such emergent of defense mechanisms related to tumor-associated acidosis can further enhance the ability of the cells to survive harsh environmental conditions.^126,127^

Acidosis can also strongly contribute to the GBM TME becoming highly immunosuppressive; such immunosuppression is in part achieved by the acidosis and hypoxia induced upregulation of the synthesis of hypoxanthine and transmembrane CD44 receptors given that adhesion between tumor cells can be reduced as a result of hypoxanthine and CD44 binding; this has profound effects on multiple components of immune cell function which could potentially be targetable for therapeutic strategies.^137,138^ Beyond its impact on immunity, acidosis in general is known to provide a protective microenvironment for dormant tumor cells that supports the survival and invasion of circulating tumor cells and promotes resistance to chemotherapy and radiotherapy. In addition, the acidic environment has been found to promote anoikis resistance via mTOR/NF-κB signaling.^139^ Whether similar mechanisms also apply to glioma cells in the brain microenvironment to support cell survival, treatment resistance, and infiltrative phenotype remains under study.

Cancer cells use the Warburg effect (aerobic glycolysis) to meet their high energy demands. However, the results of recent studies suggest that cancer cells under aerobic glycolysis can switch to oxidative phosphorylation by downregulating hypoxia-inducible factors (HIF)-1α, resulting in a more aggressive phenotype. The metabolic plasticity of tumor cells, which allows them to adjust according to the changing microenvironment, gives them a selective advantage.^140^ This plasticity has been demonstrated in a subset of the GBM cell population which were found to have both high glycolytic and oxidative phosphorylation phenotypes, a phenomenon that was leveraged to use metabolic inhibitors leading to a change in their bioenergetics state. Thus, targeting of acidosis with the use of inhibitors that target pH balance in the TME along with standard-of-care therapy is emerging as another therapeutic strategy against GBMs,^141^ a concept that is being tested in ongoing clinical trials (NCT03011671).

### Hypoxia

Another chemical environmental factor that helps cancer cells acquire resistance is hypoxia which is a fundamental driver of oncogenesis which develops when oxygen consumption demands from cell proliferation outstrips the ability of neoangiogenesis or diffusion to restore oxygenation. The hypoxic environment has been well established as a key trigger for angiogenesis, invasion, survival, and resistance to therapies in GBMs; additionally, tumor hypoxia also directly correlate with disease progression and poor prognosis in GBM patients.^142,143^ Tumor cells respond to hypoxic conditions by upregulating HIFs which in turn activate gene expression of downstream targets needed for cell survival.^144^ A change in oxygen tension (pO_2_) is sensed by prolyl-4-hydroxylase 2 (PHD2), which regulates the expression of HIFs.^145^ During normoxic conditions, PHD2 is activated in the presence of oxygen and 2-oxoglutarate and downregulates HIF-1α by hydroxylation at its Pro402 and Pro564 residues at C terminus.^144,146–148^ The hydroxylated HIF-1α binds to pVHL and is degraded by proteasome polyubiquitination.^147^ During hypoxic conditions, low oxygen tension leads to inhibition of pHD2, causing HIF-1α accumulation. The dimerization of HIF-1α and HIF-1β leads to the upregulation of proangiogenic genes, such as VEGF, fibroblast growth factor, and several others, which leads to an increase in angiogenesis, invasiveness, and tumor migration.^149^ Depending on the cellular context, PHD2 exerts both pro- and antitumor properties. In breast cancer, PHD2 has been reported to promote tumor cell migration via cancer-associated fibroblast activation. On the other hand, downregulation of PHD2 showed an antitumor effect in GBM,^148,150^ head and neck squamous cell carcinoma,^151^ and bone marrow-derived cells.^152^ Considering the significance of PHD2 in tumor progression and HIF regulation, PHD2 could be a potential therapeutic target in the treatment of GBM and other cancer types. There are 2 ongoing clinical trials targeting hypoxia using MBM-02 (Tempol) and belzutifan to inhibit HIFs in GBM patients (NCT04874506 and NCT02974738).

## Modeling the GBM TME

To enhance knowledge of the GBM TME and the development of specific treatment strategies related to TME, a variety of in vitro and in vivo preclinical models have been developed. In this section, we discuss the features and limitation of TME models (Table 1). An ideal TME model should faithfully represent the various constituents of the human GBM TME and demonstrate similar plasticity and regional variations that recapitulate human gliomas; in addition, they should adequately represent the BBB function and cell-to-cell interaction (tumor–tumor and tumor–nontumor cells).^101^ The ideal model should be similar to human glioma genetically and should adequately represent intratumoral heterogeneity along with relevant host factors.

**Table 1.** Models of Tumor Microenvironment in Gliomas

Type of Model	Features		Limitations	References
2D model		• Well-characterized human and animal cell model • Easy to maintain and manipulate • Low cost and simplified • Used in high-throughput drug screening	• Does not represent the TME adequately because of the lack of CSC-TME interactions and absence of gradient behavior for nutrient, oxygen, and pH • Prone to genomic alteration because of long-term culture	^153–155^
3D cell-based models	Spheroid-based models	• Consist of mainly spherical disorganized cancer cells • Preserve cell–cell and cell–matrix interaction • Closely represent in vivo cell behaviors such as cell morphology, proliferation, differentiation, invasion, and metabolism • Most commonly used 3D in vitro model • Easy production	• Does not represent the TME adequately as an uncontrolled cell–cell and cell–matrix interaction • Does not represent tumor heterogeneity	^156–158^
	Organoid models	• Self-organized group of cells from the same or different genetic makeup that captures genetic, microenvironmental, and histopathological characteristics of original tumors • Interactions among tumor, immune, and stem cells are preserved • Capture tumor heterogeneity and the hypoxic gradient	• Not a sustainable system, as it has large necrotic regions • Not reproducible because of its self-organized nature	^159–161^
Tissue engineering-based model	3D bioprinting	• Cells are added to the scaffold using computer assistance, with or without exogenous material • More precise than manual cell-based 3D models • Mimics organ functions and organ interactions at a certain degree	• Inadequate reproducibility, cell density control, and spatial distribution control • Does not capture the dynamic nature of the TME	^162–164^
	4D bioprinting	• Next generation for biofabrication technology to mimic the in vivo TME • Stimuli-responsive biomaterials are used that modify in a time-dependent manner • Used in drug screening and drug delivery studies, comprehension of glioma progression and therapy	• Impact of human physiology on the TME is not integrated	^165–167^
Rodent models	Xenograft	• Patient-derived cells or tissues are implanted in immunodeficient mice • Suitable for studying orthotropic and heterotrophic interactions • One of the most commonly used animal models	• Because of the lack of immune cells, the TME is not completely represented	^168^
	Syngeneic	• Immunologically competent tumor cells are implanted in immunocompetent mice • No graft -versus -host reactions • Suitable for immune studies	• Limited clinical relevance because of the lack of human glioma cells in the TME	^169^
	Humanized	• Human hematopoietic stem cells are transplanted into immunodeficient mice that lead to development of the human immune system • Suitable for patient-oriented research, such as immunotherapeutic drug development and other infectious diseases	• Variable TME response, depending on the origin of the cells used for immunocompetent and xenograft cells	^170^
	Genetically engineered Immunocompetent	• Ideal for studying genetic factors involved in the development of brain tumors • Invasive method of tumor implantation could be avoided • Limited changes in the TME because of the intact BBB	• Development of lethal genetic abnormalities • Limited mutation in models limits representation of the human TME in full complexity	^171^

BBB, blood–brain barrier; TME, tumor microenvironment.

Based on cellular composition, these models are categorized as cell-based, tissue engineering-based and animal models. In vitro 2D models are the most used ones because of the ease of use and maintenance. However, they lack the 3D representation of the various TME cellular components and do not have a BBB.^156,157^ Simpler 3D models using cellular aggregates or neurospheres provide an improved representation of glioma and the TME that allow studies of simple cell–cell interactions and spatial relationships and in the case of patient-derived glioma stem-like cell spheres, also represent human tumors more faithfully genetically than attached cells. Tumor cell secretions, responses to changes in nutrients, pH and oxygen, and tumor–normal cell interactions can be preliminarily studied using such models but they still do not represent the native TME and do not have aBBB.^158,162^ More recent developments have to led to matrix-assisted 3D tumor models which can be used to study drug delivery systems and soluble factor signaling. They can also be used to prescreen drugs before in vivo experiments, reducing the time and effort required and minimizes use of animals (Table 2).^158,162^

**Table 2.** Selected Active Clinical Trials Involving/Exploring the Glioma Microenvironment

Trial ID	Title	Location
NCT04781764	The Study of Microglia/Macrophages Involved Dynamic Evolution of Glioma Microenvironment and the Function and Visualization of Targeted Molecules of Glioma	Hushan Hospital Fudan University, China
NCT04729959	A Safety Run-In and Phase II Study Evaluating the Efficacy, Safety, and Impact on the Tumor Microenvironment of the Combination of Tocilizumab, Atezolizumab, and Fractionated Stereotactic Radiotherapy in Recurrent Glioblastoma	Peking University Third Hospital, China
NCT04461938	Characterization of Metabolic Changes in Glioma Tumor Tissue Induced by Transient Fasting (ERGO3)	Goethe University, Germany
NCT04606316	A Phase Ib Clinical Trial to Evaluate Early Immunologic Pharmacodynamic Parameters Following Neoadjuvant Anti-PD-1 (Nivolumab), or the Combination of Anti-PD-1 Plus Anti-CTLA-4 (Nivolumab Plus Ipilimumab) in Patients with Surgically Accessible Glioblastoma	Dana-Farber Cancer Center, USA
NCT05053880	A Phase 1b/2a Study of ACT001 and Anti-PD-1 in Patients with Surgically Accessible Recurrent Glioblastoma Multiforme	The University of Texas MD Anderson Cancer Center, USA
NCT03673787	Ice-CAP: A Phase I Trial of Ipatasertib in Combination with Atezolizumab in Patients with Advanced Solid Tumours with PI3K Pathway Hyperactivation	Institute of Cancer Research, UK
NCT04656535	A Multi-Center Phase 0/I Trial of Anti-TIGIT Antibody AB154 in Combination with Anti-PD-1 Antibody AB122 for Recurrent Glioblastoma	Yale University, USA

A more relevant 3D model uses organotypic slice cultures which are derived directly from the brain tissue and hence closely mimics the in vivo system making them suitable for various brain and spine studies. Delbridge et al. found that microglia isolated from organotypic slice cultures were similar to acutely isolated adult microglia; they used this model successfully to study neuroinflammation.^172^ Recent findings have shown that neurons and glial cells are preserved in organotypic hippocampal slice cultures.^173^ Similar models have also been developed from glioma tissue in which organotypic slice cultures generated from freshly resected human or animal tumors have been successfully used to study tumor biology and therapeutic responses. Such slice cultures contain both tumor and nontumor microenvironmental cells in their native spatial distribution which provides a window to examine the intercellular relationships and the opportunity to modulate the same therapeutically in an ex vivo setting. Because of the true representation of tissue heterogeneity and microenvironmental components in these ex vivo cultures, they have become an integral part of preclinical drug testing.^174–176^ Although organotypic slice cultures have several advantages over conventional 2D and 3D models, they also have limitations including the effects of the specific culture conditions used and the lack of a functional BBB; however, this model provides the most direct data related to biology and treatment response of human tumor and TME outside of clinical studies. Another relevant model uses tissue engineering methods including microfluidic systems and tissue matrices in an effort to reconstruct the complexity of human gliomas through the so-called tumor-on-a-chip model.; these are also increasingly used to study the impact of TME components on tumor development and progression.^165–168,177^

Development of preclinical models, while useful for studying specific questions related to the GBM TME, do not fully represent the highly complex nature of TME and cell-to-cell interactions. Therefore, in vivo tumor models may be more relevant to study the TME and related cellular interactions. Most studies involve using patient-derived xenograft cells implanted in immune-compromised mice, limiting the representation of the immune component of TME. This shortcoming can partly be resolved using a syngeneic brain tumor model. However, this may lead to differences between species and not completely represent a native TME. The development of humanized mouse models, which have components of the human immune system interacting with tumor xenografts, allows a better representation of the immune TME; in addition, these models can be genetically modified to address specific biological questions. Continued optimization of such preclinical models by overcoming their various limitations is ongoing with the goal of generating a more relevant model with true fidelity to the human glioma.^168–171^

## Concluding Remarks

Recent advances in science have enhanced the understanding of the TME in GBM; however, translation of these findings to therapeutic strategies against the disease has not yet been optimally achieved and the disease prognosis remains poor. The bidirectional interaction of tumor cells with the neighboring microenvironment results in a complex multicellular system that enables tumor cell proliferation and resistance to treatments including chemotherapy, radiotherapy, and other related treatments. Recent in-depth studies have helped better understand the composition of the TME and the interaction of the TME with glioma cells. Additionally, development of robust tumor models derived from patient specimens with immune, stromal, and cancer cells have further improved preclinical target assessment and help to identify key elements for therapeutic targeting and develop novel approaches to anti-glioma therapy targeting the points of interaction between gliomas and their TME.
